# Evaluation of two different carriers in the biodegradation process of an azo dye

**DOI:** 10.1007/s40201-019-00377-8

**Published:** 2019-05-29

**Authors:** Graziely Cristina Santos-Pereira, Carlos Renato Corso, Jörgen Forss

**Affiliations:** 1 Department of Built Environment and Energy Technology, Faculty of Technology, Linnaeus University, Växjö, Sweden; 2 Department of Biochemistry and Microbiology, Institute of Biosciences, UNESP - São Paulo State University , Rio Claro, SP Brazil

**Keywords:** Decolourization, Rice husks, MBBR, Direct red 75, Textile wastewater treatment

## Abstract

**Purpose:**

The MBBR solution has been applied for the textile wastewater treatment. However, in order to develop cost-effective solutions, waste biomass can be used as carrier. Rice husks are agricultural waste which have been used as an adsorbent of dyes; besides, they can provide and sustain suitable microorganism communities for the degradation of dyes. This study aimed to evaluate the biodegradation of the azo dye Direct Red 75 in two treatment systems with different carriers.

**Methods:**

Bioreactor A was composed by an anaerobic bioreactor filled with Kaldnes K1 carriers employed in the MBBR technology and the study was performed in 2 different temperatures, 30 ± 0.5 °C and 21 ± 2 °C. Biofilter B was composed by a sequenced anaerobic-aerobic system with rice husks as carriers and this study was performed at 21 ± 2 °C. The rice husks was also employed as a source of microorganisms in both systems. Decolourization, surface area of the carriers and other parameters were analysed.

**Results:**

Biofilter B showed high rates of decolorization, mainly over 90% in all HRT tested (24, 48 and 12 h), presenting itself as a stable system, whereas Bioreactors A showed better performances with 48 h of HRT, about 85% for A at 30 ± 0.5 °C and 45% at 21 ± 2 °C. With a similar amount of carriers, analyses showed that rice husks had a much larger surface for microorganisms to grow on than Kaldnes K1.

**Conclusion:**

The Biofilter B is a worthwhile system to be investigated and applied for the decolourization of textile wastewater treatment; for instance, in developing countries.

## Introduction

With the continual growth of the human population comes an increase in the use of water, both for human use and for application in agriculture and industrial processes. Industries consume a large amount of water in their processes. According to the United Nations Educational, Scientific and Cultural Organization (UNESCO) [1], approximately 22% of the total use of water in the world is for industry and, in developing countries, 70% of the industrial residues are discharged into rivers without treatment.

The textile industries are one of those which use the most water in their processes, in addition to many chemical products such as dyes, detergents, salts and mordants. As great water consumers, these industries are also great generators of effluent, containing substances from different phases of dyeing, finishing and other processes. The pollutants found in the textile effluents are mainly persistent organic substances, such as dyes, which confer low biodegradability to the effluent [2].

Azo dyes make up the largest group of dyes (60–70%); therefore they constitute the majority of the wastewater of textile industries and are the most studied class of dye [3, 4]. Azo dye has azo groups (–N=N–) attached to aromatic rings. Besides the textile application, this class is also used in the pharmaceutical, food, leather, paper and cosmetics industries [5].

According to Wijetunga et al. [6], the dyes are continually being upgraded and replaced by compounds with improved stability and resistance to natural degradation. For this reason it is necessary to develop new technologies which can improve textile wastewater treatment.

There are various methods of treatment; however, none is completely effective and some require a combination of techniques to be efficient. For instance, the conventional aerobic processes have been insufficient to degrade most azo dyes [7, 8]. Huge investment has been made on research to improve the quality of industrial wastewater treatment in an attempt to minimize the polluting and toxic potential of the residues.

During the past 150 years, biological treatment was the most common and widely used process in wastewater treatment. It has been applied to remove organic compounds and the colour of textile effluents, due to their low cost, simple operation and maintenance [2, 3, 9]. Bacteria are performing most of the degradation in the treatment; however, fungi are also found associated in the consortia [10–14].

According to Zollinger [15], degradation of azo dyes starts with reductive cleavage of the azo group, releasing amines as metabolites. Some aromatic amines are toxic and carcinogenic to the organisms present in the environment [7, 8, 16, 17]. The complete mineralization of the dyes would be ideal for minimizing the pollution by textile effluents; however, the synthetic dyes are compounds difficult to mineralize.

Different methods to improve the biodegradation process have been evaluated by researchers all over the world. For instance, methods that use isolated species or a consortium of microorganisms [10, 11, 18, 19]; using bioreactors as UASB [6, 20, 21] and using Moving Bed Biofilm Reactor (MBBR) to evaluate dye biodegradation [22].

The MBBR technology employs floating plastic carriers, such as Kaldnes, for the growth of biofilms and provides both aerobic and anaerobic environments for the microorganisms, facilitating cooperation between these different groups [22, 23]. According to Borkar et al. [24], there are two forms of biomass in the MBBR process: suspended flocks and a biofilm attached to carriers. The MBBR solution has a higher density of microorganisms, it can be operated at high organic loads and it is less sensitive to hydraulic overloading and starving periods [24, 25].

In order to develop cost-effective solutions, waste biomasses and low cost materials can be used as carriers and, also, as alternative adsorbents for the removal of heavy metals and organic pollutants [24, 26] .

Some waste products from industrial or agricultural operations have potential as inexpensive adsorbents [27]. For instance, rice husks are agricultural waste available in excess which have been used as an adsorbent of dyes [26–29]. Moreover, Forss et al. [14], Forss et al. [30] and Türgay et al. [31] have shown that rice husks can provide and sustain suitable microorganism communities for the degradation of dyes and actual textile wastewater.

Thus, rice husks could be suitable for use as a carrier in wastewater treatment in developing countries. Those countries have many problems with textile wastewater, and rice husks are a cost-effective and readily available material. According to Schneider et al. [16], the use of azo dyes has been drastically reduced in Europe as a result of some regulations, but it is still a problem in some non-European countries.

Focusing on the utilization of microorganisms and carrier material for the degradation of azo dyes, the present study aimed to evaluate the biodegradation of the azo dye Direct Red 75 (DR75) in anaerobic and anaerobic-aerobic bioreactors with Kaldnes K1 and rice husks, respectively, as support material. The changes in the rice husks surface were also investigated during the experiment.

## Material and methods

### Dye

**Fig. 1:**
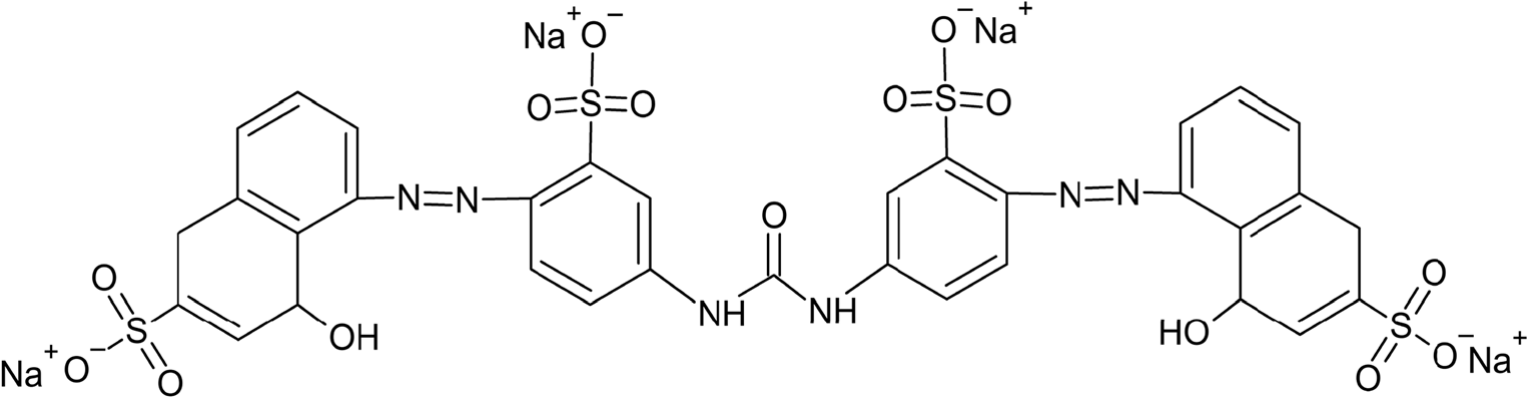
Chemical structure of the azo dye Direct Red 75

Direct Red 75 (DR75), Colour Index 25,380, CAS 2829-43-8 with molecular weight 990.79 is a direct azo dye obtained from the Sigma-Aldrich Chemical Company Inc. (Fig. 1). The dye was used without additional purification.

The culture medium contained yeast extract 2 g/l, NaCl 0.9% and DR75 dye 100 mg/l. Sodium phosphate buffer (0.2 M) was added to the culture medium in order to keep the pH around 7.0. Chemical Oxygen Demand (COD) of this synthetic wastewater was 2347 mg/L.

### Microorganisms

Rice husks from a rice processing company in Uberlândia - Minas Gerais, Brazil - were used as a source of microorganisms. The rinse water used for inoculating the microorganisms in the Bioreactor A was prepared with 250 mL of distilled water and 12 g of rice husks under stirring with magnetic bars for 30 min at 300 rpm. The rice husks were used without any preparation in the Biofilter B.

### Treatment systems

Two different treatment systems were used to analyse the azo dye biodegradation, one anaerobic treatment and another one sequential anaerobic–aerobic treatment. The bioreactors were fed continuously with a culture medium at the required rate (2 L/day for HRT of 24 h, 1 L/day for HRT of 48 h and 4 L/day for HRT of 12 h) using a peristaltic pump Watson Marlow 400, Sci-Q.

#### Bioreactor A (anaerobic bioreactor)

**Fig. 2:**
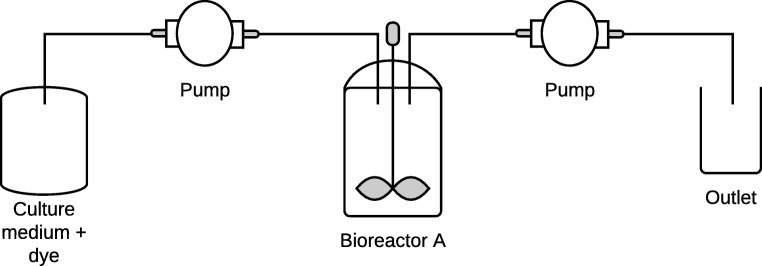
Bioreactor A diagram

Bioreactor A was a compact benchtop fermenter (Fig. 2). The temperature, pO_2_, pH and stirring were controlled by the unit control Biostat B, B Braun Biotech.

MBBR technology was applied in Bioreactor A and had Kaldnes K1, obtained from AnoxKaldnes™, as carrier material. The Kaldnes K1 is a cylinder with a cross inside and fins on the outside; the length is 700 mm, the diameter is 10 mm, the specific surface area is 690 m^2^/m^3^ total and 500 m^2^/m^3^ effective [32].

About 50% of the effective bioreactor volume was filled with the carriers. The process was anaerobic and the vessel had capacity for 2 L of culture medium. The system ran with 2 different temperatures, at the same room temperature (21 ± 2 °C) as Biofilter B and with a constant temperature at 30 ± 0.5 °C, pH around 6, constant stirring at 100 rpm and hydraulic retention time (HRT) of 24, 48 and 12 h. In order to test if the microbial growth during the experiment had developed a more efficient consortium that could compensate the HRT needed for degradation, the HRT of 12 h was tested after the HRT of 48 h as a complementary study, therefore 12 h HRT is shown after 48 h in the results in section 3.

The first 24 h there was no flow in the system to favour the growth of the microorganisms and their attachment to the carriers.

#### Biofilter B (Fixed Bed Biofilter)

**Fig. 3:**
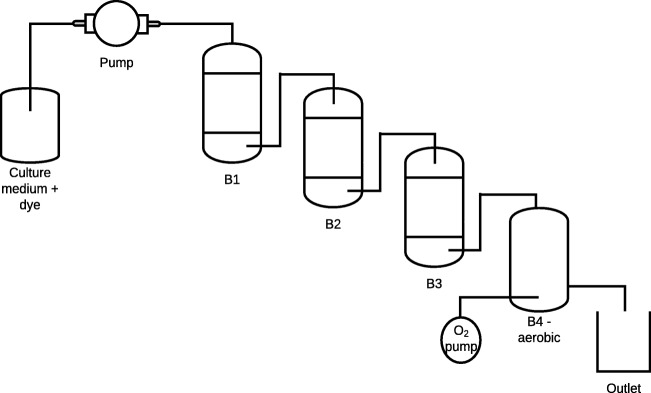
Biofilter B diagram (anaerobic bioreactors B1, B2, B3 and the aerobic bioreactor B4)

The Biofilter B consisted of three anaerobic bioreactors (B1, B2 and B3) with capacity for 350 mL of culture medium each and one aerobic bioreactor (B4) with capacity for 500 mL (Fig. 3). Each anaerobic bioreactor was filled with, approximately, 40.0 g of rice husks (dry weight) and the aerobic bioreactor with, approximately, 4.0 g of rice husks (dry weight).

The rice husks were used as carrier material and source of microorganisms. The process was performed without stirring at room temperature (21 ± 2 °C), pH around 6 in the anaerobic bioreactors and around 8 in the aerobic bioreactor. The retention time was also 24, 48 and 12 h.

Another aerobic bioreactor, with greater volume, was added to the system after the HRT change from 48 to 12 h. The addition occurred due to the results of COD removal to keep the HRT of 48 h in the aerobic reactor part although the anaerobic bioreactors were adjusted to 12 h. The aim was to enhance the COD removal.

### Analysis

All samples were centrifuged before the analyses in a centrifuge Sigma 3 K10 in 7000 rpm for 7 min.

#### UV-VIS spectrophotometry

The samples were analysed by UV-VIS spectrophotometry using a LAMBDA 35 UV-VIS spectrophotometer Perkin-Elmer and the UV WinLab software. Samples were taken and scanned from 200 nm to 800 nm three times a week.

The absorbance of the chromophore group was used to monitor the decolourization. The chromophore wavelength to the medium culture with DR75 was 517 nm. The decolourization of the dye was evaluated using the following Eq. (1):

1 $$ \frac{\mathrm{Dye}\kern.1em \mathrm{solution}\kern0.17em \mathrm{absorbance}\kern0.17em \mathrm{Sample}\kern0.17em \mathrm{absorbance}\kern.1em }{\mathrm{Dye}\kern.1em \mathrm{solution}\kern0.17em \mathrm{absorbance}}\kern.1em \mathrm{X}\kern.1em 100 $$

#### Aromatic amines

LC/MS and LC/MS/MS analyses were performed to detect aromatic amines possibly generated in the dye biodegradation process. The samples were collected from the outlets of the bioreactors A 30 ± 0.5 °C, B1, B2, B3 and B4 in the 5th week and bioreactor A 30 ± 0.5 °C and B4 in the 9th and 11th week of treatment. These samples were derivatized by the addition of benzyl chloroformate in order to detect the substrate by parental ion scanning, specifically for detection of the benzyl group. Benzyl chloroformate (4 μL) was added to 100 μl 1 ppm amines in 1:2:2 water:methanol:acetonitrile solution. The reaction mixture was kept at room temperature for 5 min even though the reaction is almost instant. No further dilution was performed before analysis by LC/MS.

LC/MS method: The samples were separated by a 3 min linear gradient from 95% mobile phase A (95% H_2_O + 5% Acetonitrile +0.1% NH_3_) to 95% mobile phase B (Acetonitrile) with 0.8 ml/min flow rate at 70 °C on an Acquity UPLC BEH C18 (1.7 μm, 2.1 × 50 mm) column.

Three aromatic amines were analysed as reference substances to verify the method. The reference substances, 2,4-toluene diamine (TDA), sulfanilic acid and 4-amino-3-hydroxi-1-naphthalenesulfonic acid were used to check the reaction and detection conditions and DR75 was used as reference azo dye.

#### COD, organic acids, nitrogen, phosphate and Orto-phosphate

COD was measured over the weeks to evaluate the organic matter degradation by the microorganisms.

COD, organic acids such as acetic acid and butanoic acid, nitrogen, phosphate and orto-phosphate concentrations were determined using Hach colourimetry test cuvettes (LCK014, LCK114, LCK365, LCK238, LCK350, and LCK 049, respectively), Hach-Lange LT 200 thermostat and Hach-Lange DR 2800 spectrophotometer.

COD and Organic acids analyses were monitored weekly while nitrogen, phosphate and orto-phosphate were carried out with the medium culture before to enter in the system and the final samples of the Bioreactors A and the aerobic bioreactor (B4) outlet in the last day of treatment.

#### Surface area of rice husks

Surface area of rice husks before and after the treatment was measured using Brunauer–Emmett–Teller (BET) method. Samples of rice husks before the dye treatment, rice husks after treatment washed with water and with ethanol 70% were evaluated. The washed husks were also shaken for 3 min, approximately.

The samples were grounded and dried under vacuum at 85 °C overnight in Micrometrics VacPrep 061. The analysis of specific surface area was done using Micrometrics TriStar 3000 Analyzer. The measurements were taken under N_2_ adsorption at liquid nitrogen temperature.

#### Pore size of rice husks

Mesopores and a portion of the macropores of the rice husks, from 1.7 up to 300 nm, were measured by Barrett-Joyner-Halenda (BJH) method. The samples were prepared and analysed as described in the previous section for surface area analysis.

Macropores from 100 nm up to 50,000 nm were characterized by the mercury intrusion porosimetry (MIP) method using a Micromeritics’ AutoPore IV 9500 Series. The samples were dried at 85 °C before the analysis. The porosity was measured using a 5 bulb solid penetrometer of 1 mL, stem volume 0.392 ml, penetrometer constant 10.79 ul/pF and pressure 0–30,000 psia.

## Results

### Decolourization

Both systems developed well, Bioreactors A reached a stable decolourization more quickly than Biofilter B; however, system B performed a much better decolourization. Since Biofilter B was filled with rice husks, a natural material, the initial process of decolourization occurred also by adsorption of the dye molecules.

**Fig. 4:**
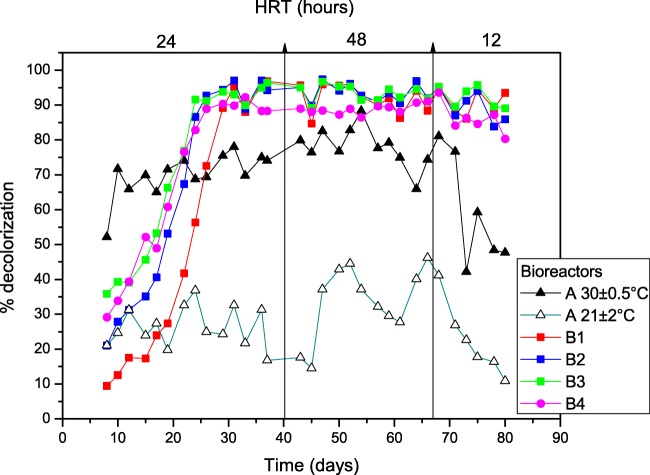
Percentage of decolourization in the anaerobic Bioreactors A (30 ± 0,5 °C and 21 ± 2 °C), and Biofilter B (21 ± 2 °C) composed by the anaerobic bioreactors B1, B2, B3 and the aerobic bioreactor B4 with 24, 48 and 12 h of HRT

The systems were evaluated at hydraulic retention times (HRT) of 24, 48 and 12 h. As can be seen in Fig. 4, the stabilization time for the Bioreactors A, with treating capacity of 2 L, was shorter than the Biofilter B, with treating capacity of 1.55 L in total, and the decolourization rates fluctuated according to the change in the HRT. After changing the HRT in Bioreactor A at 30 ± 0.5 °C to 48 h, the percentage of decolourization increased from about 67.9% to 78.3%. When reducing the HRT to 12 h, the decolourization rates decreased to 45%, approximately.

On the other hand the percentages of decolourization in Biofilter B were higher than Bioreactors A after 22 days. Even with lower treating capacity, Biofilter B was considerably more efficient with respect to the decolourization than the Bioreactors A, regardless of the temperature set for the treatment.

The bioreactors B1, B2, B3 and B4 were assessed in an attempt to follow the development and performance of Biofilter B. Table 1 shows the performance of each reactor, regarding the percentage of decolourization of the incoming solution (outlet from previous reactor).

**Table 1:** Performance of the separate reactors in Biofilter B during the experiment, showing the percentage of decolourization in bioreactors B1, B2, B3 and B4 with 24, 48 and 12 h of retention time

	Week	B1 (%)	B2-B1 (%)	B3-B2 (%)	B4-B3 (%)
24 h	1	17.50	17.82	10.50	6.48
	3	72.59	5.16	−0.55	−3.41
	6	95.93	−1.49	1.19	−8.01
48 h	7	92.48	1.06	0.49	−1.79
	8	86.30	2.78	−2.36	−3.80
	9	95.13	−1.24	2.54	−5.40
12 h	10	94.47	−5.10	5.78	−2.41
	11	93.49	0.23	8.20	−11.79
	12	83.79	2.95	0.83	−3.74

The system HRT was 24, 48 and 12 h, i.e., 6, 12 and 3 h, respectively for each reactor in the biofilter. The negative results show that absorbance was slightly higher in the outlet than the inlet (from a very low level) when taking samples. Thus, it can be seen that the microbial society developed well and the degradation mainly took place in the first reactor.

In addition to the decolourization measurements, the possible presence of aromatic amines as metabolites of the treatment was investigated by a LC/MS technique. It had been verified that this method could detect the test substances. However, the concentration of aromatic amines in the samples analysed was too low to be detected by the instrument.

### Chemical analyses

Nitrogen and phosphorus were evaluated because they are related to the capacity of the microorganisms to grow and biodegrade the dye. The concentration of carbon, total nitrogen, phosphate and orto-phosphate must be in the proper proportions to enable cell growth without nutrient limitation.

The orto-phosphate is directly available to the microorganism and the concentration can be evaluated to ensure that there will be enough phosphorus to support the microbial growth. The concentrations of phosphate and orto-phosphate in the culture medium at the beginning of the treatment were 8.63 mg/l and 24.8 mg/l, respectively. After the treatment, the phosphate concentrations were 20.9 mg/l in Bioreactor A and 20 mg/l in Biofilter B and the orto-phosphate concentrations were 28.6 mg/l in Bioreactor A and 27.6 mg/l in Biofilter B.

The total nitrogen concentration at the beginning of the treatment was 234 mg/l and after the treatment it was 226 mg/l in Bioreactor A and 191.4 mg/l in Biofilter B. Considering the rate C:N:P as 250:5:1 in anaerobic treatment, 100:5:1 in aerobic treatment [33, 34] and the medium culture COD of 2347 mg/l, the concentrations of carbon, nitrogen and phosphate were in the proper proportions to enable microbial growth without nutrient limitation.

The concentration of organic acids was measured to follow the anaerobic activity. In Biofilter B that was sequential anaerobic-aerobic, organic acids were produced during the anaerobic digestion (dye degradation), and then consumed in the aerobic reactor. Table 2 shows the percentage of COD removal and concentration of organic acids of the samples.

**Table 2:** % of COD removal from culture medium and organic acids concentration in the dye biodegradation process

Bioreactor	COD removal (%)			Organic acids ^a^ (mg/l)		
	HRT 24 h	HRT 48 h	HRT 12 h	HRT 24 h	HRT 48 h	HRT 12 h
A 30 °C	15.38	19.55	12.89	625.40	527.25	697.00
A 21 ± 2 °C	6.15	18.05	11.16	558.71	585.00	668.00
B1	7.33	8.36	17.28	690.00	727.00	710.00
B2	18.41	14.89	17.21	703.00	666.00	796.00
B3	19.28	25.61	13.78	634.00	559.50	748.00
B4	55.62	71.76	62.44 ^b^	304.00	152.48	285.65 ^b^

a) Organic acids concentration for the culture medium is 280 mg/l

b) HRT for B4 was 48 h due to the insertion of another aerobic bioreactor with larger volume

The percentage of COD removal for the Bioreactors A, anaerobic, was lower than the bioreactor B3, the last anaerobic reactor of Biofilter B. The most efficient COD removal in Biofilter B was found in the aerobic bioreactor B4. Despite the decolourization rates being above 80% for Biofilter B, the percentage of COD removal is low in the anaerobic biofilters.

The decolourization took place when the azo bond was broken by the action of the microorganisms [35]; however, it does not mean that they were mineralized, it is just that the molecules became smaller. For this reason, the decolourization rates might be high while the COD removal efficiencies are low in the anaerobic systems. Although, it can also be noted that released microbial biomass, metabolites from the rice husks and the organic acids produced also give rise to COD.

According to Dafale et al. [36], the usual low efficiency of COD removal by conventional techniques has been overcome by the development of anaerobic-aerobic systems and the results achieved in this research support this statement.

The HRT could also influence the COD removal. Both systems had better removal of COD with increased HRT as can be seen in Table 2.

Moreover, after changing the HRT from 48 to 12 h, the percentage of COD removal decreased in B4, meanwhile, the organic acids concentration increased. In light of these results, the HRT was increased to 48 h only in the aerobic bioreactor (B4), by the insertion of another aerobic bioreactor with greater volume, whereas the anaerobic bioreactors remained with HRT of 12 h, in order to enhance the COD removal.

Volatile suspended solids (VSS) were not analysed because no precipitate was visible in the centrifuged samples.

### Rice husks surface area and pore size

Rice husks were used as support material for the growth of microorganisms in the Biofilter B as well as the Kaldnes K1 that was used in Bioreactor A. The surface area of both carriers was compared because even though they are so distinct, they were used with the same purpose in the present study.

Since the rice husk is an organic material, the surface area after treatment was observed to check the effects of the microbial activity on the rice husks. Therefore, the rice husk’s surface area before and after treatment was assessed along with the pore size. The results are presented in Table 3.

**Table 3:** Surface area and pore analysis of rice husks before and after biological treatment

Method	BET	BJH		Mercury intrusion porosimetry	
Sample	Surface Area (m^2^/g)	Total pore area (m^2^/g)	Total pore volume (m^2^/g)	Total pore area (m^2^/g)	Total pore volume (m^2^/g)
Rice husks before treatment	1.00 ± 0.02	1,15 ± 0,04	0,0097 ± 0,001	1,23 ± 0.01	0.123 ± 0,004
Rice husks washed with H_2_O	1.58 ± 0.18	2,47 ± 0,10	0,0110 ± 0,0004	1,38 ± 0.08	0.134 ± 0,004
Rice husks washed with ethanol 70%	2.06 ± 0.09	2,95 ± 0,09	0,0488 ± 0,05	1,34 ± 0.08	0.131 ± 0,002

The differences among the samples could be assigned to the action of microorganisms.

### Carrier evaluation

According Ødegaard et al. [32] the total specific surface area of the carriers Kaldnes K1 is 690 m^2^/m^3^, the effective specific surface area, i.e., the area that actually has growth of biofilm, is 500 m^2^/m^3^, the bulk carriers is 1030 pieces/l and the density of the HDPE is 0.95 ± 0.02 g/cm^3^ (9.5 × 10^6^ g/m^3^). The Bioreactors A were filled with 1 L of Kaldnes K1, which corresponds to 132 g of carriers, hence, the surface area of the these carriers was 0.69 m^2^.

Bioreactors B1, B2 and B3 were filled with, approximately, 40.0 g of rice husks (dry weight). Since Biofilter B, with total volume of 1.05 L, was composed of 3 anaerobic bioreactors, the total amount of the rice husks was 120 g (dry weight). The surface area for rice husks, measured by BET method, before treatment was 1 m^2^/g (Table 3). Thus, the total surface area available was 0.69 m^2^ for the plastic carriers and 120 m^2^ for the rice husks. Therefore, Biofilter B had the largest surface area of the systems evaluated, providing a larger area for biofilm establishment.

## Discussion

In order to design an efficient and sustainable bioreactor solution for textile wastewater treatment, good decolourization performance is required; however there are several other parameters to be evaluated. The present study verified some important factors in the biodegradation process.

Both systems investigated have distinct microbial activity since one is anaerobic and the other one is anaerobic-aerobic. Parameters such as diverse carrier material and temperature were tested as well as aromatic amines generation, COD removal, nitrogen, phosphate and organic acids concentration. However, some parameters can be compared in order to find a better setup for a future treatment system encompassing the best characteristics of each process. For instance, the temperature of Bioreactor A was maintained at 30 ± 0.5 °C and at room temperature whereas Biofilter B was maintained only at room temperature. It was expected that the decolourization in Bioreactor A at 30 ± 0.5 °C would be higher than Biofilter B because of the temperature. According to Angelova et al. [37], which evaluated the temperature effect on bacterial azo bond reduction, the decolourization rates increase as the temperature also increases until a certain limit for bacteria. On the other hand, the decolourization rates of Biofilter B were better than Bioreactor A in both temperatures, indicating that even with lower temperature, Biofilter B was more efficient as regards decolourization.

Another difference between Bioreactor A and Biofilter B is the effect of HRT in the decolourization. The HRT was changed twice to evaluate the biodegradation process over time and to verify which HRT would be more advantageous to these treatment systems. The system was initiated with 24 h of HRT, then it was changed to 48 h after 40 days, approximately, and it was changed once more to 12 h after almost 70 days of the treatment beginning.

When the HRT was increased to 48 h and afterwards it was decreased to 12 h, the decolourization in bioreactors B1, B2 and B3 did not have a substantial variation unlike Bioreactor A. Thus, system A seems to be more sensitive to HRT changes than Bioreactor B. Therefore, despite it taking a longer time to stabilize, Biofilter B was more efficient at decolorizing the dye solution than Bioreactor A. As textile industries are concerned with the colour and COD removal of the wastewater, it is of interest to them to use a system of wastewater treatment that will be able to remove the colour and increase the percentage of COD removal, as much as possible, even if the system needs a certain time to stabilize. More important in the long run are factors such as durability, resilience to fluctuations in loads and starvation periods, parameters where a biofilm system has clear advantages.

HRT of 24 and 48 h was set for the whole treatment system. However, if each bioreactor from Biofilter B is analysed separately, the HRT will be 6 and 12 h/bioreactor, respectively. Although, after 30 days, B2 and B3 have shown high decolourization rates as well as B1, B1 was still mainly responsible for decolourization in Biofilter B in this period, after system stabilization. Furthermore, B1 was the first bioreactor to receive the culture medium rich in nutrients, which likely favoured its microorganisms. Moreover, at steady state, B1 seems to have developed a microbial consortium that efficiently decolorizes the incoming flow of azo dyes.

Forss et al. [30] also analysed the biodegradation of dyes, Reactive Black 5 and Reactive Red 2, in two bioreactors filled with rice husks. A total of 80% decolourization was reached after 28.4 h of HRT, i.e., 14.2 h for each bioreactor. Additionally, a recent review by Vikrant et al. [38] compiled different bioremediation studies of bioremediation of azo dyes, however, it is complex to compare different dyes, it can be noted that the studies performed use in average 24-60 h to degrade 100 mg/l. Therefore, with HRT of 6 h/bioreactor, Bioreactor B has proven to be an interesting alternative to dye biodegradation and should be further assessed, so this technique can be possibly applied in the future for textile wastewater treatment.

Although the present study has evaluated the degradation process of one dye considering different carriers, previous studies of Forss et al. [14] have shown that actual textile wastewater can affect degradation differently. Moreover, an actual wastewater stream might change content depending on different process flows. Thus, when designing a biofilter, it is preferable to have enough capacity so the biofilter can buffer these fluctuations in load. It can be stated conclusively that the system may benefit from having the HRT longer than the minimum of 6 h/bioreactor, so that a consortium can degrade a wider variety of dyes and other chemicals in the wastewater. The most cost-effective system could be consisted of two anaerobic bioreactors, since Bioreactor B3 had no noticeable activity during the decolourization process, as it would still have enough capacity to deal with variations in flow and composition of wastewater. The COD removal was also affected by the HRT changes. According to Işık and Sponza [8], about 80% COD and 91% colour removal efficiencies were obtained at a HRT of 100 h in a sequential anaerobic/aerobic reactor system. After the HRT decreased to 6 h, the COD removal efficiencies decreased to 29.4%. Considering that the COD removal in the present study, with HRT of 24 h, was about 15% in Bioreactor A and 56% in Biofilter B, it is concluded that COD removal might be better with a longer HRT, since the COD removal, with HRT of 48 h, was about 20% in A and 72% in B.

The HRT along with the concentration of oxygen in the bioreactors has influenced the COD removal in the systems. The aerobic process was more efficient for COD removal than the anaerobic process.

The colour removal was provided by the reductive cleavage of the dye bond, which used a carbon source as an electron donor through anaerobic decolourization under reductive conditions in bioreactors [39].

Decolourization means that the azo bonds of DR75 were broken, thus colour was removed from the wastewater and aromatic amines might have been generated. However, the metabolites of DR75 were still remained in the solution during anaerobic treatment being degraded later by the aerobic treatment [40]. Therefore COD removal was lower in the anaerobic treatment. Analysing azo dye treatment, Ozdemir et al. [41] observed that the aeration of the last of four compartments of the anaerobic baffled reactor (ABR) increased the COD removal efficiency from approximately 89% to 97%. This result was achieved due to aerobic degradation of the residual COD [41] as occurred in the B4 aerobic bioreactor (Table 2).

Furthermore, the aromatic amines generated by the reductive cleavage of azo bonds, as sulfanilic acid, can be oxidized to organic acids [42]. For this reason the concentration of aromatic amines could be low in the bioreactors’ samples, hence, not detected by the LC/MS technique.

The organic acid concentration is higher in anaerobic bioreactors because a large fraction of substrate carbon is converted to fatty acids and a smaller fraction, less than 30%, is converted to cell mass while in aerobic fermentation, about 50% of substrate carbon is incorporated into cells and about 50% of it is used as an energy source [43].

The chemical analysis of the biodegradation process in Bioreactor A and Biofilter B showed that the conditions for microbial growth were favourable. The concentrations of phosphate and orto-phosphate after treatment were higher than before, indicating there was enough phosphorus for the microorganisms or even a surplus of phosphorus. According Wade [44], the concentration of cell phosphorus increases in the first phases of bacteria growth and decreases until the end of a generation. Considering that bacterial DNA can be released to the environment, as demonstrated by Turk et al. [45], the higher concentration of phosphorus measured in this study could be related to the dissolved DNA from the microorganisms.

Observing the final nitrogen concentration, it is possible to propose that besides the nitrogen consumed by the microorganisms; a fraction of microorganisms’ residue was released into the wastewater.

The difference between the samples of rice husks before treatment and washed with water and ethanol can be assigned to the action of microorganisms. The rice husks are composed of several compounds such as cellulose, hemicellulose and lignin [46]. Paethanom and Yoshikawa [47] investigated the rice husks and characterized them, in weight percentage (wt.%) dry basis, containing carbon 37.9 wt.%, nitrogen 0.4 wt.% and hydrogen 3.9 wt.%. Hence, the rice husks could likely be partly degraded by the microorganisms and, consequently, new pores could be created, increasing the total pore area of macropores and mesopores. Macropores above 50,000 nm and micropores were not assessed by mercury intrusion porosimetry because the results were distorted. The measurements above 50,000 nm have indicated voids while the micropores were destroyed by the high pressure employed for this technique. According Chesson et al. [48], micropores of biological materials are more likely to be closed ended; therefore, it is difficult to support the different pressures applied to them.

As the rice husks are so robust, in order to protect the grain, they can be possibly used in the system for a long time. This study had the system running for more than 3 months, as shown in Fig. 4, and the husks performed well with no need to change them.

The adsorption capacity of rice husk, rice husk ash as well as its functional groups of adsorbents has been assessed for several researchers [26, 49–52]. Despite that, there is still a lack of information about the surface of the rice husks and the present study can help to fill this gap, since it is one of the first pieces of research to consider the interaction of microorganisms and the surface of rice husks.

MBBR processes use specially designed plastic carriers as support material where biomass can attach and grow on the surface forming a biofilm [53].

Rice husk is an organic material not specifically designed for wastewater treatment, however, microorganisms often find it easy to attach to natural materials and it has functioned as a carrier and source of microorganisms in dye biodegradation [31, 54].

Moreover, several studies have shown that biofilm formation enhances decolourization and it is favoured by the presence of a carrier at the beginning of the treatment [22, 55–57].

Even though the two systems had a similar amount of carriers in grams, using approximately the same volume and space, the available surface in the bioreactors differed. Additionally, since the rice husks are organic material and the MBBR process uses polyethylene carriers, the microorganisms could also partly consume the husks as a carbon and nitrogen source. Clearly this difference results in an improved degradation performance and function in Biofilter B. Since the systems had different carriers, the decolourization capacity can be related to the difference between the nature and surface area of the support material and the microorganisms that formed the biofilm.

## Conclusion

The sequenced anaerobic-aerobic treatment system with rice husks as carrier and source of microorganisms presented the best decolourization rates. The COD was best removed by the aerobic bioreactor suggesting that textile wastewater is best treated by a sequenced anaerobic-aerobic system.

The rice husks proved to be more efficient in treatment than the Kaldnes K1 tested. With a similar amount of carriers, rice husks had a much larger surface for microorganisms to grow on. Hence, a biofilter solution with rice husks will be more effective per m^3^.

These findings support that it is possible to design a cost-effective system, since rice husks are cheaper than MBBR solutions, and it could be applied for the textile wastewater treatment in developing countries. Certainly, the rice husk is a versatile material because in addition as acting as a carrier material, it is also a source of microorganisms.

Furthermore, the Fixed Bed Biofilter (Biofilter B) evaluated in this study presented high stability after the adaptation period for the microorganisms. Within 22 days the decolourization rates were stable against the change of HRT. Furthermore, the system could handle the fluctuations even after 80 days of treatment and with no need to change the rice husks.

Therefore, the Fixed Bed Biofilter proposed by this study is a worthwhile sequenced anaerobic/aerobic treatment system to be further investigated and applied for the decolourization of textile wastewater treatment; for instance, in developing countries.

## References

[1] UNESCO (2003). Water for people, water for life - UN world water development report (WWDR).

[2] Fu ZM, Zhang YG, Wang XJ (2011). Textiles wastewater treatment using anoxic filter bed and biological wriggle bed-ozone biological aerated filter. Bioresour Technol.

[3] Hunger K. Industrial dyes: chemistry, properties, Applications. 1st ed. Hunger K, editor. Weinheim, Germany: WILEY-VCH; 2003.

[4] van der Zee FP, Bisschops IAE, Blanchard VG (2003). The contribution of biotic and abiotic processes during azo dye reduction in anaerobic sludge. Water Res.

[5] Telke A, Kalyani D, Jadhav J (2008). Kinetics and mechanism of reactive red 141 degradation by a bacterial isolate *Rhizobium radiobacter* MTCC 8161. Acta Chim Slov.

[6] Wijetunga S, Li X-F, Jian C (2010). Effect of organic load on decolourization of textile wastewater containing acid dyes in upflow anaerobic sludge blanket reactor. J Hazard Mater.

[7] Almeida EJ, Corso CR (2014). Comparative study of toxicity of azo dye Procion red MX-5B following biosorption and biodegradation treatments with the fungi *Aspergillus niger* and *Aspergillus terreus*. Chemosphere.

[8] Işık M, Sponza DT (2008). Anaerobic/aerobic treatment of a simulated textile wastewater. Sep Purif Technol.

[9] Dafale N, Rao NN, Meshram SU (2008). Decolorization of azo dyes and simulated dye bath wastewater using acclimatized microbial consortium – biostimulation and halo tolerance. Bioresour Technol.

[10] Corso CR, Almeida EJ, Santos GC (2012). Bioremediation of direct dyes in simulated textile effluents by a paramorphogenic form of *Aspergillus oryzae*. Water Sci Technol.

[11] Santos GC, Corso CR (2014). Comparative Analysis of Azo Dye Biodegradation by *Aspergillus oryzae* and *Phanerochaete chrysosporium*. Water Air Soil Pollut.

[12] Corso CR, Almeida ACM (2009). Bioremediation of Dyes in Textile Effluents by *Aspergillus oryzae*. Microb Ecol.

[13] El-Rahim WMA, Moawad H, Abdel Azeiz AZ (2017). Optimization of conditions for decolorization of azo-based textile dyes by multiple fungal species. J Biotech.

[14] Forss J, Lindh MV, Pinhassi J (2017). Microbial biotreatment of actual textile wastewater in a continuous sequential Rice husk biofilter and the microbial community involved. PLoS One.

[15] Zollinger H (1991). Color Chamistry: syntheses, properties and applications of organic dyes and pigments.

[16] Schneider K, Hafner C, Jager I (2004). Mutagenicity of textile dye products. J Appl Toxicol.

[17] Brüschweiler BJ, Merlot C (2017). Azo dyes in clothing textiles can be cleaved into a series of mutagenic aromatic amines which are not regulated yet. Regul Toxicol Pharmacol.

[18] Yang Q, Li C, Li H (2009). Degradation of synthetic reactive azo dyes and treatment of textile wastewater by a fungi consortium reactor. Biochem Eng J.

[19] Tang B, Chen Q, Bin L (2018). Insight into the microbial community and its succession of a coupling anaerobic-aerobic biofilm on semi-suspended bio-carriers. Bioresour Technol.

[20] Baêta BEL, Aquino SF, Silva SQ (2012). Anaerobic degradation of azo dye Drimaren blue HFRL in UASB reactor in the presence of yeast extract a source of carbon and redox mediator. Biodegradation.

[21] Alvarez LH, Arvizu IC, García-Reyes RB (2017). Quinone-functionalized activated carbon improves the reduction of congo red coupled to the removal of p-cresol in a UASB reactor. J Hazard Mater.

[22] Calderón K, Martín-Pascual J, Poyatos JM (2012). Comparative analysis of the bacterial diversity in a lab-scale moving bed biofilm reactor (MBBR) applied to treat urban wastewater under different operational conditions. Bioresour Technol.

[23] Almstrand R, Persson F, Daims H (2014). Three-dimensional stratification of bacterial biofilm populations in a moving bed biofilm reactor for Nitritation-Anammox. Int J Mol Sci.

[24] Borkar RP, Gulhane ML, Kotangale AJ (2013). Moving bed biofilm reactor – a new perspective in wastewater treatment. IOSR J Environ Sci Toxicol Food Technol.

[25] Sipma J, Osuna B, Collado N (2010). Comparison of removal of pharmaceuticals in MBR and activated sludge systems. Desalination.

[26] Safa Y, Bhatti HN (2011). Biosorption of direct Red-31 and direct Orange-26 dyes by rice husk: application of factorial design analysis. Chem Eng Res Des.

[27] Chuah TG, Jumasiah A, Azni I (2005). Rice husk as a potentially low-cost biosorbent for heavy metal and dye removal: an overview. Desalination.

[28] Lakshmi UR, Srivastava VC, Mall ID (2009). Rice husk ash as an effective adsorbent: evaluation of adsorptive characteristics for indigo carmine dye. J Environ Manag.

[29] Vadivelan V, Kumar KV (2005). Equilibrium, kinetics, mechanism, and process design for the sorption of methylene blue onto rice husk. J Colloid Interface Sci.

[30] Forss J, Pinhassi J, Lindh M (2013). Microbial diversity in a continuous system based on rice husks for biodegradation of the azo dyes Reactive Red 2 and Reactive Black 5. Bioresour Technol.

[31] Türgay O, Ersöz G, Atalay S (2011). The treatment of azo dyes found in textile industry wastewater by anaerobic biological method and chemical oxidation. Sep Purif Technol.

[32] Ødegaard H, Gisvold B, Strickland J (2000). The influence of carrier size and shape in the moving bed biofilm process. Water Sci Technol.

[33] Ammary BY (2004). Nutrients requirements in biological industrial wastewater treatment. Afr J Biotechnol.

[34] Thompson LJ, Gray V, Lindsay D (2006). Carbon : nitrogen : phosphorus ratios influence biofilm formation by *Enterobacter cloacae* and *Citrobacter freundii*. J Appl Microbiol.

[35] Saratale RG, Saratale GD, Chang JS (2011). Bacterial decolorization and degradation of azo dyes: a review. J Taiwan Inst Chem Eng.

[36] Dafale N, Wate S, Meshram S (2010). Bioremediation of wastewater containing azo dyes through sequential anaerobic–aerobic bioreactor system and its biodiversity. Environ Rev.

[37] Angelova B, Avramova T, Stefanova L (2008). Temperature effect on bacterial azo bond reduction kinetics: an Arrhenius plot analysis. Biodegradation..

[38] Vikrant K, Giri BS, Raza N (2018). Recent advancements in bioremediation of dye: Current status and challenges. Bioresour Technol.

[39] Lim CK, Aris A, Neoh CH (2014). Evaluation of macrocomposite based sequencing batch biofilm reactor (MC-SBBR) for decolorization and biodegradation of azo dye acid Orange 7. Int Biodeterior Biodegradation.

[40] van der Zee FP, Villaverde S (2005). Combined anaerobic-aerobic treatment of azo dyes--a short review of bioreactor studies. Water Res.

[41] Ozdemir S, Cirik K, Akman D (2013). Treatment of azo dye-containing synthetic textile dye effluent using sulfidogenic anaerobic baffled reactor. Bioresour Technol.

[42] Shuler ML, Kargi F (2002). Bioprocess engineering: basic concepts.

[43] Jonstrup M, Kumar N, Murto M (2011). Sequential anaerobic–aerobic treatment of azo dyes: Decolourisation and amine degradability. Desalination.

[44] Wade HE (1952). Variation in the phosphorus content of *Escherichia coli* during cultivation. J Gen Microbiol.

[45] Turk V, Rehnstam A-S, Lundberg E (1992). Release of bacterial DNA by marine Nanoflagellates, an intermediate step in phosphorus regeneration. Appl Environ Microbiol.

[46] Kumar PS, Ramakrishnan K, Kirupha SD (2010). Thermodynamic and kinetic studies of cadmium adsorption from aqueous solution onto rice husk. Braz J Chem Eng.

[47] Paethanom A, Yoshikawa K (2012). Influence of pyrolysis temperature on Rice husk char characteristics and its tar adsorption capability. Energies..

[48] Chesson A, Gardner PT, Wood TJ (1997). Cell wall porosity and available surface area of wheat straw and wheat grain fractions. J Sci Food Agric.

[49] Daffalla SB, Mukhtar H, Shaharun MS (2010). Characterization of adsorbent Developed from Rice husk: effect of surface functional group on phenol adsorption. J Appl Sci.

[50] Nakbanpote W, Goodman BA, Thiravetyan P (2007). Copper adsorption on rice husk derived materials studied by EPR and FTIR. Colloids Surf A Physicochem Eng Asp.

[51] Jiang Z, Hu D (2019). Molecular mechanism of anionic dyes adsorption on cationized rice husk cellulose from agricultural wastes. J Mol Liq.

[52] Sawasdee S, Jankerd H, Watcharabundit P (2017). Adsorption of dyestuff in household-scale dyeing onto rice husk. Energy Procedia.

[53] Habouzit F, Hamelin J, Santa-Catalina G (2014). Biofilm development during the start-up period of anaerobic biofilm reactors: the biofilm archaea community is highly dependent on the support material. Microb Biotechnol.

[54] Hoellein TJ, Tank JL, Kelly JJ (2010). Seasonal variation in nutrient limitation of microbial biofilms colonizing organic and inorganic substrata in streams. Hydrobiologia..

[55] Ratcliffe M, Rogers C, Merdinger M (2006). Treatment of high strength chemical industry wastewater using moving bed biofilm reactor (MBBR) and powdered activated carbon (PAC) technology. Proc Water Environ Fed.

[56] Verhagen P, De Gelder L, Hoefman S (2011). Planktonic versus biofilm catabolic communities: importance of the biofilm for species selection and pesticide degradation. Appl Environ Microbiol.

[57] Zhu Y, Zhang Y, Ren HQ (2015). Physicochemical characteristics and microbial community evolution of biofilms during the start-up period in a moving bed biofilm reactor. Bioresour Technol.

